# Short-term Forecasts of the COVID-19 Epidemic in Guangdong and Zhejiang, China: February 13–23, 2020

**DOI:** 10.3390/jcm9020596

**Published:** 2020-02-22

**Authors:** Kimberlyn Roosa, Yiseul Lee, Ruiyan Luo, Alexander Kirpich, Richard Rothenberg, James M. Hyman, Ping Yan, Gerardo Chowell

**Affiliations:** 1 Department of Population Health Sciences, School of Public Health, Georgia State University, Atlanta, GA 30302, USA; ylee97@gsu.edu (Y.L.); rluo@gsu.edu (R.L.); akirpich@gsu.edu (A.K.); rrothenberg@gsu.edu (R.R.); gchowell@gsu.edu (G.C.); 2 Department of Mathematics, Center for Computational Science, Tulane University, New Orleans, LA 70118, USA; mhyman@tulane.edu; 3 Infectious Disease Prevention and Control Branch, Public Health Agency of Canada, Ottawa, ON K1A 0K9, Canada; ping.yan@canada.ca

**Keywords:** COVID-19, coronavirus, China, real-time forecasts, phenomenological models, sub-epidemic model

## Abstract

The ongoing COVID-19 epidemic continues to spread within and outside of China, despite several social distancing measures implemented by the Chinese government. Limited epidemiological data are available, and recent changes in case definition and reporting further complicate our understanding of the impact of the epidemic, particularly in the epidemic’s epicenter. Here we use previously validated phenomenological models to generate short-term forecasts of cumulative reported cases in Guangdong and Zhejiang, China. Using daily reported cumulative case data up until 13 February 2020 from the National Health Commission of China, we report 5- and 10-day ahead forecasts of cumulative case reports. Specifically, we generate forecasts using a generalized logistic growth model, the Richards growth model, and a sub-epidemic wave model, which have each been previously used to forecast outbreaks due to different infectious diseases. Forecasts from each of the models suggest the outbreaks may be nearing extinction in both Guangdong and Zhejiang; however, the sub-epidemic model predictions also include the potential for further sustained transmission, particularly in Zhejiang. Our 10-day forecasts across the three models predict an additional 65–81 cases (upper bounds: 169–507) in Guangdong and an additional 44–354 (upper bounds: 141–875) cases in Zhejiang by February 23, 2020. In the best-case scenario, current data suggest that transmission in both provinces is slowing down.

## 1. Introduction

The ongoing epidemic of a novel coronavirus illness (COVID-19) began in Hubei Province, China, in December 2019 and continues to cause infections in multiple countries, threatening to become a pandemic. However, the bulk of the associated morbidity and mortality is still concentrated within the province of Hubei, China. As of 13 February 2020, there have been 59,907 cumulative cases, including 1368 deaths, reported globally with 48,206 cases reported in Hubei alone [1]. To control the epidemic, the Chinese government has enacted a range of social distancing strategies, such as city-wide lockdowns, screening measures at train stations and airports, active case finding, and isolation of suspected cases. The numbers of cases and deaths continue to accumulate every day. However, the transmission appears to be slowing down outside Hubei due to strict lockdowns combined with isolation and quarantine measures [1,2,3].

While the transmission potential of this novel coronavirus can reach high values [4,5], the epidemiological features of COVID-19 are still unclear, and changes in reporting of cases and deaths complicate the analysis of the epidemic. For instance, the case definition has been revised over time, and, as of 12 February 2020, reported cases of the disease incorporate clinically suspected cases in addition to laboratory-confirmed cases, which led to a noticeable increase in cases in the province of Hubei on 13 February 2020. These additional cases are likely historical cases that occurred days or weeks earlier. In the absence of additional information, this change in reporting obscures the true underlying epidemic trajectory, especially in Hubei, and complicates the inference of epidemiological parameters, such as the effective reproduction number, and the calibration of mechanistic transmission models that rely on data after 12 February 2020.

Earlier work has shown that phenomenological growth models, including the sub-epidemic growth model, can capture the empirical patterns of past epidemics and are useful to generate short-term forecasts of the epidemic trajectory in real time. These approaches are especially useful when the epidemiological data are limited [6,7,8,9]. Real-time short-term forecasts generated from such models can be useful to guide the allocation of resources that are critical to bring the epidemic under control. In this paper, we use dynamic models to generate 5-day and 10-day ahead forecasts of the cumulative reported cases in the provinces of Guangdong and Zhejiang, China.

## 2. Methods

### 2.1. Data

We use data from the National Health Commission of China which reports the cumulative cases for 34 provinces, including municipalities, autonomous regions, and special administrative regions [1]. We collected reported case data each day at 12 pm (GMT-5) from the initial date of reporting, 22 January 2020, to 13 February 2020. We then forecasted the trajectory of the epidemic in the provinces of Guangdong and Zhejiang, which have exhibited a high burden of COVID-19. We did not fit the data or forecast the epidemic in Hubei, as the recent change in the reporting criteria resulted in a significant jump in reported cases on 13 February 2020. The impact of the new reporting criteria in this province will require a detailed analysis before the data can be fit by our current models.

### 2.2. Models

We use three phenomenological models that have been previously applied to various infectious disease outbreaks including other respiratory illnesses, such as severe acute respiratory syndrome (SARS) and pandemic influenza [10,11], and to this current outbreak [12]. The generalized logistic growth model (GLM) and the Richards model extend the simple logistic growth model with an additional scaling parameter [9,11,13]. We also apply a sub-epidemic model, which accommodates complex epidemic trajectories, such as multiple peaks and sustained or damped oscillations, by assembling the contribution of inferred overlapping sub-epidemics [10]. Appendix A includes a detailed description of the models and their parameters.

### 2.3. Short-Term Forecasts

We calibrate each of the models to the daily case counts reported for Guangdong and Zhejiang provinces. We fit the model to the “incidence” curve while presenting the cumulative curves for visualization. Reported data are available beginning 22 January 2020, so the calibration period includes daily data from 22 January – 13 February 2020. We estimate the best-fit solution for each model using nonlinear least squares fitting, a process that yields the set of model parameters that minimizes the sum of squared errors between the model and the data. The initial conditions are set to the first data point, and initial parameter estimates can be found in Table S1.

We use a parametric bootstrap approach to generate uncertainty bounds around the best-fit solution assuming a Poisson error structure; detailed descriptions of this method are provided in references [9,14]. We refit the models to each of the M = 200 datasets generated by the bootstrap approach, resulting in M best-fit parameter sets that are used to construct the 95% confidence intervals for each parameter. Further, each model solution is used to generate m = 30 additional simulations extended through a 10-day forecasting period. We construct the 95% prediction intervals for forecasts with these 6000 (M × m) curves.

## 3. Results

**Figure 1:**
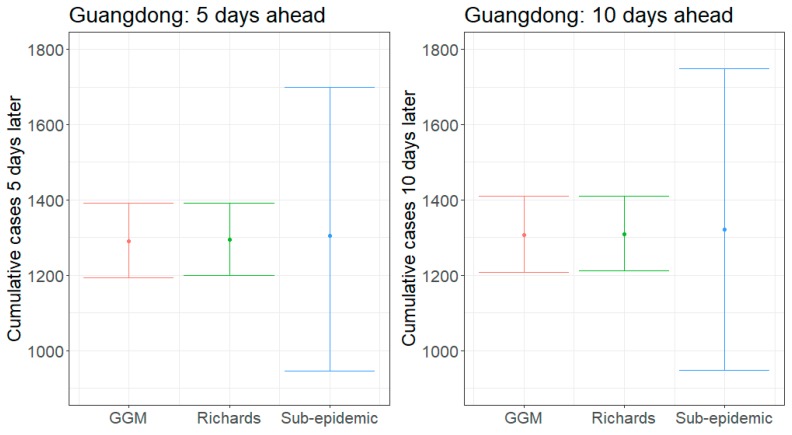
Forecasting results of 5- and 10-day ahead estimates of cumulative reported case counts for Guangdong, China, generated on 13 February 2020. The dots are the mean estimates for each model, and the hinge lines represent the 95% prediction intervals.

**Figure 2:**
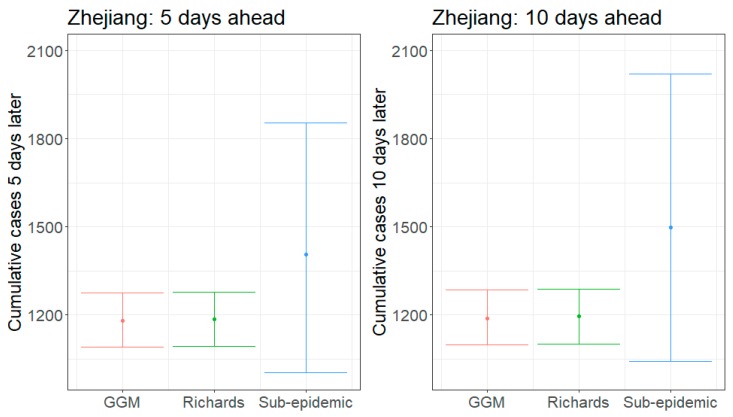
Forecasting results of 5- and 10-day ahead estimates of cumulative reported case counts for Zhejiang, China, generated on 13 February 2020. The dots are the mean estimates for each model, and the hinge lines represent the 95% prediction intervals.

**Figure 3:**
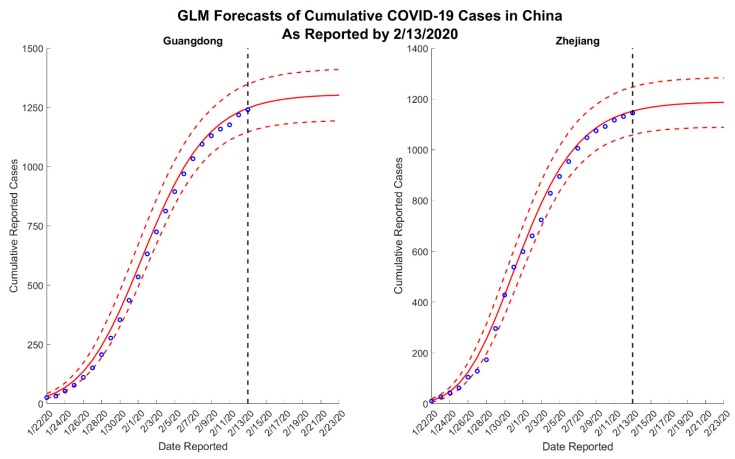
Ten-day ahead logistic growth model forecasts of cumulative reported COVID-19 cases in Guangdong and Zhejiang, China, generated on 13 February 2020. The blue circles correspond to the cumulative cases reported up until 13 February 2020; the solid red lines correspond to the mean model solution; the dashed red lines depict the 95% prediction intervals; and the black vertical dashed line separates the calibration and forecasting periods.

**Figure 4:**
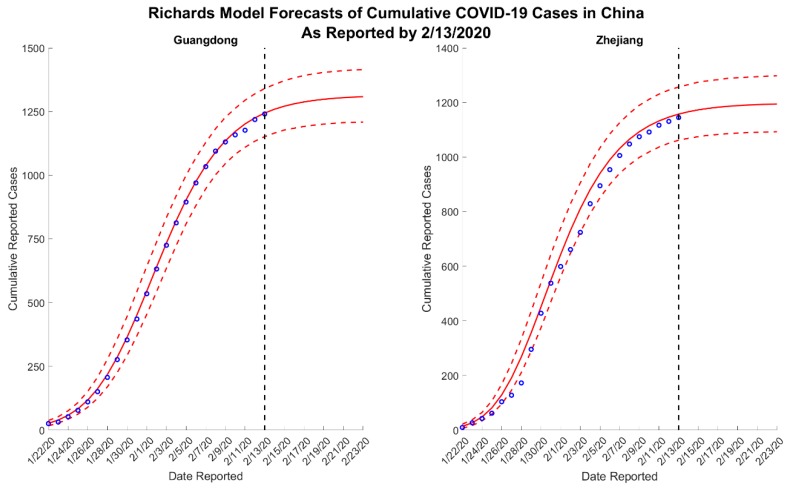
Ten-day ahead Richards model forecasts of cumulative reported COVID-19 cases in Guangdong and Zhejiang, China, generated on 13 February 2020. The blue circles correspond to the cumulative cases reported up until 13 February 2020; the solid red lines correspond to the mean model solution; the dashed red lines depict the 95% prediction intervals; and the black vertical dashed line separates the calibration and forecasting periods.

**Figure 5:**
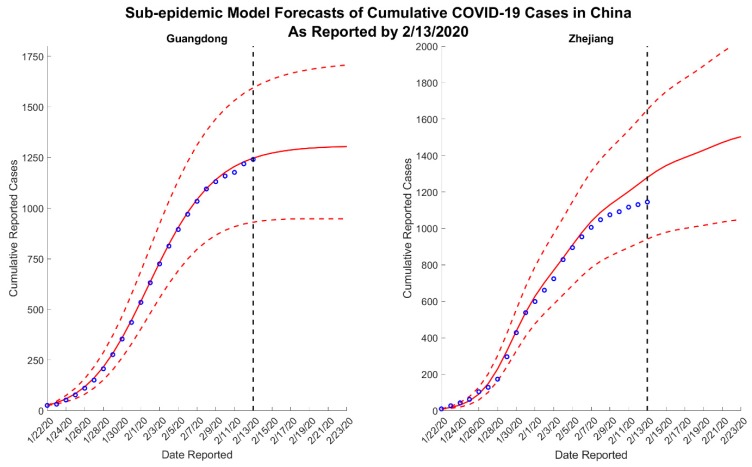
Ten-day ahead sub-epidemic model forecasts of cumulative reported COVID-19 cases in Guangdong and Zhejiang, China, generated on 13 February 2020. The blue circles correspond to the cumulative cases reported up until 13 February 2020; the solid red lines correspond to the mean model solution; the dashed red lines depict the 95% prediction intervals; and the black vertical dashed line separates the calibration and forecasting periods.

We present results for 5- and 10-day forecasts generated on 13 February 2020 for the provinces of Guangdong and Zhejiang, China. Figure 1 and Figure 2 contain the estimated ranges of cumulative case counts from 5- and 10-day forecasts for Guangdong and Zhejiang, respectively. 10-day ahead forecasts from each model with the reported calibration data are shown in Figure 3, Figure 4 and Figure 5.

### 3.1. Guangdong

Our 5-day average forecasts for Guangdong are nearly equivalent across the three models, ranging from 1290–1304 cumulative reported cases (Figure 1). As of 13 February 2020, Guangdong has 1241 reported cases [1], so forecasts predict an additional 49–63 cases in the next 5 days. Upper bounds (UB) of 95% prediction intervals for both the GLM and Richards model suggest that up to 1392 cases could accumulate, while the sub-epidemic prediction intervals are substantially wider and include up to 1699 cases; this translates to an additional 151–458 additional cases by 18 February 2020.

The 10-day forecasts suggest very little increase from the 5-day forecasts, especially for those predicted by the GLM and Richards model (Figure 1). Average 10-day forecasts predict between 1306–1322 cumulative cases with upper bounds ranging from 1410–1748 cases. These forecasts suggest that an additional 65–81 cases (UB: 169–507) will be reported by 23 February 2020.

### 3.2. Zhejiang

Average 5-day forecasts from the GLM and Richards model are nearly equivalent for Zhejiang (1181 and 1186, respectively), while the sub-epidemic model predicts an average of 1405 cumulative cases (Figure 2). The sub-epidemic model also has significantly higher upper bounds, suggesting the possibility of up to 1853 cases, while the GLM and Richards only predict up to 1276 and 1279, respectively. As of 13 February 2020, Zhejiang has a total cumulative reported case count of 1145 [1]; therefore, the models are predicting an additional 36–260 cases in the next five days (UB: 131–708).

Our 10-day forecasts from the GLM and Richards model show little increase in cases from 5 to 10 days ahead; however, the sub-epidemic model forecasts increase significantly during this time (Figure 2). 10-day forecasts across the models predict 1189–1499 cumulative cases, on average, with upper bounds ranging from 1286–2020 cases. This corresponds to an additional 44–354 (UB: 141–875) cases in Zhejiang by 23 February 2020.

## 4. Discussion

We present timely short-term forecasts for reported cases of COVID-19 in Guangdong and Zhejiang, China. Based on data reported up to 13 February 2020, the models predict 65–81 additional cases (UB: 169–507) in Guangdong and 44–354 (UB: 141–875) additional cases in Zhejiang by 23 February 2020. Overall, our forecasts suggest that the epidemics in these two provinces continue to slow down.

Across all forecasts, the GLM and Richards model provide comparable mean estimates and prediction intervals, while the sub-epidemic model forecasts exhibit significantly greater uncertainty (Figure 1 and Figure 2). While the mean estimates for Guangdong are nearly equivalent across all three models, the mean estimates generated by the sub-epidemic model are significantly higher for Zhejiang. Both the GLM and Richards models predict that the provinces are nearing the end of the epidemic (Figure 3 and Figure 4). However, forecasts from the sub-epidemic model, which accommodates more complex trajectories, suggest a longer epidemic wave (Figure 5). Specifically, the sub-epidemic model forecasts for Zhejiang suggest additional smaller sub-epidemics have yet to occur, resulting in higher estimates of cumulative case counts.

While we do not know the true underlying epidemic trajectory, it is reasonable to assume that the sub-epidemic forecasts better capture the uncertainty for the next 10 days. The fluctuating case definition may partially explain the slowing down observed in the data that result in the GLM and Richards model predicting extinction. The kink in the Zhejiang data may suggest a case definition change around 6 February 2020, which would partially explain a decrease in the new daily cases reported. The slowing in cases after 6 February is apparent in both Guangdong and Zhejiang. This pattern must be interpreted with caution; it is not entirely clear whether this is a true decline in transmission, or if it is an artificial decline due to the changing case definition. Therefore, the sub-epidemic model forecasts likely better capture both possibilities. Additionally, on 14 February 2020, China officially reported that 1716 healthcare workers have been infected with the disease, suggesting disease amplification in healthcare settings as has been previously documented for past SARS and Middle East respiratory syndrome (MERS) outbreaks [15]. This analysis does not account for the greater potential for transmission in healthcare settings. Finally, we note that our forecasts are not sensitive to the last data point used to calibrate the models (13 February 2020), despite the most recent change in case reporting (Tables S2 and S3).

In conclusion, while our models predict the outbreaks in Guangdong and Zhejiang have nearly reached extinction, our forecasts need to be interpreted with caution given the unstable case definition and reporting patterns. Thus, we point readers to the sub-epidemic model predictions specifically, which suggest that another smaller wave of cases is in process. If the observed decline in case incidence is true, the predictions likely reflect the impact of the social distancing measures implemented by the Chinese government. In the best-case scenario, the model forecasts based on the current data suggest that transmission in both provinces is slowing down.

## Supplementary Materials

The following are available online at https://www.mdpi.com/2077-0383/9/2/596/s1, Table S1: Initial parameter estimates and estimation bounds for the model fitting process to calibrate each of the models to the data reported up until 13 February 2020, Table S2: Guangdong: GLM, Richards, and sub-epidemic model parameter estimates, mean squared error (MSE) for the best-fit solution, and prediction interval (PI) coverage, Table S3: Zhejiang: GLM, Richards, and sub-epidemic model parameter estimates, mean squared error (MSE) for the best-fit solution, and prediction interval (PI) coverage.

## Appendix A

### Appendix A.1. GLM

The generalized logistic growth model (GLM) extends the simple logistic growth model with a scaling of growth parameter *p* that accommodates sub-exponential growth patterns [16,17,18,19]. The GLM is defined by the differential equation

$$C^{\prime }\left(t\right)\;=\;rC{\left(t\right)}^{p}\left(1-\frac{C\left(t\right)}{K}\right)$$

where *C(t)* is the cumulative cases at time *t, r* is the early growth rate, *p* is the scaling of growth parameter, and *K* is the carrying capacity and final epidemic size. Values of *p* = 1 correspond to exponential growth, *p* = 0 represents constant growth, and 0 < *p* < 1 defines sub-exponential growth.

### Appendix A.2. Richards Model

The Richards model also extends the simple logistic growth model through a scaling parameter, *a,* that measures the deviation from the symmetric simple logistic curve [9,11,13]. The Richards model is defined by the differential equation

$$C\prime \left(t\right)\;=\;rC\left(t\right)\left(1-{\left(\frac{C\left(t\right)}{K}\right)}^{a}\right)$$

where *C(t)* represents the cumulative case count at time *t*, *r* is the growth rate, *K* is the final epidemic size, and *a* is a scaling parameter.

### Appendix A.3. Sub-Epidemic Model

While the GLM and Richards model only accommodate s-shaped dynamics, the sub-epidemic wave model supports complex epidemic trajectories by aggregating asynchronous sub-epidemics. For this approach, we assume that the observed curve is the aggregate of multiple overlapping sub-epidemics, where each sub-epidemic is modeled using the GLM [10]. An epidemic wave composed of n overlapping sub-epidemics is modeled as

$$C_{i}\prime \left(t\right)\;=\;rA_{i-1}\left(t\right)C_{i}{\left(t\right)}^{p}\left(1-\frac{C_{i}\left(t\right)}{K_{i}}\right)$$

where *C_i_(t)* is the cumulative number of infections in sub-epidemic *i* (*i* = 1, …, *n*), *K_i_* is the size of the *i^th^* sub-epidemic, and the growth rate *r* and scaling parameter *p* are the same across sub-epidemics [10]. Further, when *n* = 1, the model returns to the single-equation GLM as presented above.

The timing of onset for each consecutive sub-epidemic is modeled with a regular structure, such that the (*i* + 1)th sub-epidemic is triggered when the cumulative case count of sub-epidemic *i*, *C_i_(t)*, exceeds the threshold *C_thr_*. The (*i* + 1)th sub-epidemic begins before the *i^th^* sub-epidemic reaches extinction. The size of consecutive sub-epidemics (*K_i_*) is modeled such that the size declines exponentially for each subsequent sub-epidemic, where

$$K_{i}\;=\;K_{0}e^{-q\left(i-1\right)}$$

*K*_0_ is the size of the first sub-epidemic (*K*_1_ = *K*_0_), and *q* is the rate of decline, where *q* = 0 corresponds to no decline. The total final epidemic size is

$$K_{tot}\;=\;\overset{n_{tot}}{\underset{i\;=\;1}{\sum }}K_{0}e^{-q\left(i-1\right)}\;=\;\frac{K_{0}\left(1-e^{-qn_{tot}}\right)}{1-e^{-q}}$$

where *n*_tot_ is the finite number of overlapping sub-epidemics, calculated as

$$n_{tot}\;=\;-\frac{1}{q}\ln\left(\frac{C_{thr}}{K_{0}}\right)+1$$
