# Supplementary material for: Short-term Forecasts of the COVID-19 Epidemic in Guangdong and Zhejiang, China: February 13–23, 2020
Source: J Clin Med. 2020 Feb 22;9(2):596. doi: 10.3390/jcm9020596 (PMC7073898; doi:10.3390/jcm9020596)
Supplement: Supplementary file 1 [file jcm-09-00596-s001.pdf]

Supplemental Table 1. Initial parameter estimates and estimation bounds for the model fitting process to calibrate each of the models to the data reported up until 13 February 2020.

<table>
<tr><th>GLM</th><th>r</th><th colspan="2">p</th><th colspan="2">K</th></tr>
<tr><td></td><td>0.8 [0, 10]</td><td colspan="2">1.0 [0, 1]</td><td colspan="2">10000 [0, 1000000]</td></tr>
<tr><th>Richards</th><th>r</th><th colspan="2">a</th><th colspan="2">K</th></tr>
<tr><td></td><td>0.8 [0, 10]</td><td colspan="2">1.0 [0, 10]</td><td colspan="2">10000 [0, 1000000]</td></tr>
<tr><th>Sub-epidemic</th><th>r</th><th>p</th><th colspan="2">q</th><th>K_0</th></tr>
<tr><td></td><td>0.3 [0, 10]</td><td>0.9 [0, 1]</td><td colspan="2">0.1 [0, 10]</td><td>*Based on total cases at time of calibration</td></tr>
</table>

Supplemental Table 2. Guangdong: GLM, Richards, and sub-epidemic model parameter estimates, mean squared error (MSE) for the best-fit solution, and prediction interval (PI) coverage. Mean parameter estimates are presented with the 95% confidence intervals obtained from the *M* bootstrap solutions. Estimates of q are not presented for the sub-epidemic model for Guangdong, as only one sub-epidemic was predicted, and q was not utilized.

**GLM**	***r* (95% CI)**	***p* (95% CI)**	***K* (95% CI)**	**MSE**	**PI coverage (%)**
2/12/2020<br>2/13/2020	0.66 (0.45, 1.0)<br>0.69 (0.44, 1.07)	0.88 (0.80, 0.94)<br>0.87 (0.79, 0.94)	1305 (1202, 1379)<br>1309 (1222, 1389)	37.63<br>36.95	86.36<br>86.96
**Richards**	***r* (95% CI)**	***a* (95% CI)**	***K* (95% CI)**	**MSE**	**PI coverage**
2/12/2020<br>2/13/2020	0.45 (0.36, 0.61)<br>0.45 (0.35, 0.61)	0.59 (0.37, 0.84)<br>0.59 (0.37, 0.83)	1307 (1232, 1396)<br>1306 (1234, 1386)	36.95<br>35.87	90.91<br>86.96
**Sub-epidemic**	***r* (95% CI)**	***p* (95% CI)**	**K_0 (95% CI)**	**MSE**	**PI coverage**
2/12/2020<br>2/13/2020	0.65 (0.46, 0.87)<br>0.65 (0.46, 0.88)	0.88 (0.82, 0.94)<br>0.88 (0.82, 0.94)	1300 (1220, 1380)<br>1310 (1230, 1380)	37.46<br>35.68	90.91<br>91.30

Supplemental Table 3. Zhejiang: GLM, Richards, and sub-epidemic model parameter estimates, mean squared error (MSE) for the best-fit solution, and prediction interval (PI) coverage. Mean parameter estimates are presented with the 95% confidence intervals obtained from the *M* bootstrap solutions.

<table>
<tr><th>GLM</th><th>r (95% CI)</th><th>p (95% CI)</th><th colspan="2">K (95% CI)</th><th>MSE</th><th>PI coverage (%)</th></tr>
<tr><td>2/12/2020</td><td>1.53 (1.06, 2.09)</td><td>0.76 (0.69, 0.82)</td><td colspan="2">1182 (1121, 1254)</td><td>411.22</td><td>59.09</td></tr>
<tr><td>2/13/2020</td><td>1.57 (1.10, 2.18)</td><td>0.75 (0.68, 0.82)</td><td colspan="2">1192 (1128, 1260)</td><td>393.76</td><td>65.22</td></tr>
<tr><th>Richards</th><th>r (95% CI)</th><th>a (95% CI)</th><th colspan="2">K (95% CI)</th><th>MSE</th><th>PI coverage</th></tr>
<tr><td>2/12/2020</td><td>1.10 (0.62, 1.86)</td><td>0.25 (0.13, 0.45)</td><td colspan="2">1191 (1123, 1258)</td><td>395.89</td><td>60.87</td></tr>
<tr><td>2/13/2020</td><td>1.14 (0.72, 2.10)</td><td>0.23 (0.12, 0.39)</td><td colspan="2">1197 (1125, 1272)</td><td>378.71</td><td>59.09</td></tr>
<tr><th>Sub-epidemic</th><th>r (95% CI)</th><th>p (95% CI)</th><th>K_0 (95% CI)</th><th>q (95% CI)</th><th>MSE</th><th>PI coverage</th></tr>
<tr><td>2/12/2020</td><td>0.58 (0.55, 0.65)</td><td>0.99 (0.97, 1.0)</td><td>693 (634, 752)</td><td>0.45 (0.25, 0.65)</td><td>281.98</td><td>59.09</td></tr>
<tr><td>2/13/2020</td><td>0.60 (0.56, 0.66)</td><td>0.99 (0.97, 1.0)</td><td>735 (675, 795)</td><td>0.57 (0.38, 0.75)</td><td>278.48</td><td>56.52</td></tr>
</table>
